# Trends in histological subtypes of cutaneous melanoma in the Netherlands from 1989 to 2023 – Influence of sunlight exposure and overdiagnosis

**DOI:** 10.1177/03008916251388862

**Published:** 2026-01-18

**Authors:** Catharina C. van Niekerk, André L.M. Verbeek, Erik Brummelkamp, J. Hans D.M. Otten, J. Hans M.M. Groenewoud, Michelle M. van Rossum, Jessie J.J. Gommers

**Affiliations:** 1 Department IQ Health, Radboud University Medical Center, Nijmegen, the Netherlands; 2 Department of Dermatology, Radboud University Medical Center, Nijmegen, the Netherlands; 3 Department of Medical Imaging, Radboud University Medical Center, Nijmegen, the Netherlands

**Keywords:** Cutaneous melanoma, histological subtype, increased incidence, overdiagnosis, birth cohort

## Abstract

**Introduction:**

Melanoma incidence has continued to increase over the past decades with unchanged mortality. Different sunshine exposures across the generations complicate the interpretation of the trends. It is further suggested that half of the rising incidence rate is caused by overdiagnosis. We compared the time trends in the major but not all sunlight-related histological subtypes of cutaneous melanoma in the Netherlands in 1989-2023.

**Methods:**

With data from the Netherlands Cancer Registry, trends were assessed for superficial spreading melanoma (SSM, n=93,067), lentigo maligna melanoma (LMM, n=26,237), and nodular melanoma (NM, n=15,658). Numbers and rates were analysed by age, calendar period, and birth cohort for people born in 1925-1985. To study significant changes across the years, the joinpoint regression model was used. For the projection of new cases to 2043, we applied the Nordpred method.

**Results:**

During the past 25 years, age-standardised incidence rates for melanoma in situ increased ninefold, for invasive melanoma threefold. Significant rises were noted for SSM and LMM (fourfold) and NM (twofold). The successively born cohorts showed consistent increases in incidence rate for SSM and LMM, but to a lesser degree for NM. Around 2030, the incidence rates for all types of melanomas are expected to be highest, at approximately 100 per 100,000 population.

**Conclusions:**

The incidence of melanoma showed a steady increase from 1989 onwards. This was before early detection (that may cause overdiagnosis), was put into clinical practice. Birth cohort analysis of trends in histological subtypes will further contribute to the quantification of overdiagnosis.

## Introduction

For many countries and global regions, rising incidence rates of cutaneous melanoma have been well-documented over the past decades, but with little change or even decline in mortality.^1−4^ There is clear evidence that the steady increase in the occurrence of cutaneous melanoma has been the result of sunbathing habits with more intermittent and intense ultraviolet radiation sunlight exposure, especially during childhood.^5^ This, and survival considerations, have prompted the importance of primary prevention and early detection.^6,7^

Trends in decreased mortality are likely due to improved treatment, clinical prognosis and early detection. But in the context of rising melanoma incidence, the possibility of overdetection and diagnosis of indolent pathology by diagnostic scrutiny has been raised.^8^ Others prefer to use the term overdiagnosis, which refers to the detection of asymptomatic disease that would not have otherwise become clinically apparent during a patient’s life.^9,10^ Correlational studies have been designed attempting to quantify increased diagnostic scrutiny of early lesions with the potential of overdiagnosis.^11,12^ A recent scoping review suggests that half of the rising incidence rates are caused by overdiagnosis.^13^ An elegant attempt to underline the possibility and extent of overdiagnosis was the Italian geographic-temporal correlation study on incidence and biopsy rates by Bucchi et al.^14^ Although the authors used good documentation on skin biopsies and melanoma diagnosis and strong associations, they had to conclude that the increase in the use of biopsy may also be an effect of the underlying increase in disease rates, and not only its cause.

To unravel the complexities of trends in melanoma incidence further, we propose to consider trends in histological subtypes of cutaneous melanoma. The three most common subtypes are superficial spreading melanoma (SSM), lentigo maligna melanoma (LMM), and nodular melanoma (NM).^15^ The SSM and especially the LMM formations have a clear link with cumulative sun damage, which is assumed to be less likely in NM.^16^ In general, excess incidence rates by diagnostic scrutiny and overdiagnosis would be expected in all types, whereas larger numbers caused by increased exposure to ultraviolet radiation are likely to happen for the sun-related subtypes of SSM and LMM, but less in LM.

In this study, we aim to depict age-specific epidemiological curves by calendar period and birth cohort. Epidemiological comparisons are made for melanoma death, melanoma in situ, invasive melanoma, and three major histological subtypes for the light-skinned Dutch population between 1989 and 2023.

## Methods

### Sources of data

Data of all patients diagnosed with primary cutaneous melanoma between 1989 and 2023 in the Netherlands were received from the Netherlands Comprehensive Cancer Organization (IKNL). IKNL has been responsible for the Netherlands Cancer Registry since 1989.^17^ The registration uses data from the Dutch nationwide pathology archive containing the summaries of all pathology reports with patient and tumour characteristics. Topography and morphology are coded in accordance with the International Classification of Diseases for Oncology, third edition (ICD-O-3).^18^

In this study, we used primary data from IKNL including sex, year of birth, age at diagnosis, year of diagnosis, melanoma in situ or invasive melanoma, and morphology, i.e., seventeen different histological subtypes or classifications for melanoma. Because of small numbers, patients under the age of 20 were not selected. Age- and sex-specific population and mortality data (ICD-10 category 2.1.11) were retrieved from the publicly available Statistics Netherlands database (via statline.cbs.nl).

### Statistical methods

Statistical analysis was performed with SAS V.9.3 (SAS Institute), and graphical presentations using Microsoft Office Excel 365. The primary outcome was the trend in incidence rates of melanoma over the time period 1989–2023. As a secondary outcome metric, mortality rates over the same period were analysed. Rates were calculated as the ratio of the number of cases and population per 100,000 persons. Incidence and mortality rates were standardised for age by the direct method using the standard European population file EU-27 plus EFTA 2011−2030. Time changes in the age-standardised rates were further examined with the Joinpoint Regression Program, version 4.7.^19^ Joinpoints (moments at which significant changes in annual incidence rates occur) were determined by modelling regression lines before and after the joinpoints. In addition, the estimated annual percent change (EAPC) of the rates in the specific time segments was calculated. Next, numbers and rates were analysed by age, calendar period, and birth cohort for people born in 1925-1985.

To predict the future numbers of new cases and the age-standardised incidence rates for the periods ahead from 2024 to 2040, the Nordpred package in R was applied to the (goodness-of-fit) observations of the most recent five-year periods. To develop more realistic projections, and under the assumption that the current rates will not remain the same forever, we attenuated the model’s coefficients to estimate future incidence by 25%, 50% and 75% in the second, third, and fourth prediction period until 2040, respectively.

As the focus of this study was on patterns of epidemiological curves over time, and a comprehensive pilot study revealed only minor sex differences (data not presented), we mainly present results for men and women combined.

## Results

### Demographics

Table 1 shows the basic numbers and average rates of death and newly diagnosed patients for invasive and in situ melanoma during the period 1989−2023. The number of deaths comprised 21,762, and the number of patients 182,987. The relative frequency of melanoma death was higher for men than women, 57% vs. 43%, whereas for incidence reverse figures were observed, 46% vs. 54%. The age-standardised incidence of invasive and in situ melanoma combined has been steadily increasing from 19.5 per 100,000 population in 1989 to 73.1 per 100,000 in 2023. For invasive melanoma separately, the estimated annual percent change was 3.9%, and for melanoma in situ 6.6%. The major histological subtypes of invasive melanoma were SSM comprising 93,067 (51%) patients, LMM with 26,237 (14%) persons, and NM with 15,658 (9%) individuals. The annual increase in incidence was 4.9%, 4.7%, and 1.5%, respectively.

**Table 1.** Number and average rate of melanoma death, melanoma incidence and three histological subtypes in the Netherlands for people aged 20 and over between 1989 and 2023.

	Melanoma death	Melanoma incidence			Histological subtypes of invasive melanoma		
Aggregate data from 1989 to June 2023		Total	Invasive melanoma	Melanoma in situ	SSM, superficial spreading melanoma	LMM, lentigo maligna melanoma	NM, nodular melanoma
Number of persons Men Women	21,762 12,328 (57%) 9434 (43%)	182,987 83,457 (46%) 99,530 (54%)	138,037 63,868 (46%) 74,169 (54%)	44,950 19,589 (44%) 25,361 (56%)	93,067 41,806 (45%) 51,261 (55%)	26,237 12,125 (46%) 14,112 (54%)	15,658 8057 (51%) 7601 (49%)
Age in year, M (SD) Men Women	65.8 (15.7) 65.1 (15.0) 66.7 (16.5)	60.1 (16.0) 62.0 (15.1) 58.6 (16.6)	59.0 (16.2) 60.8 (15.3) 57.3 (16.7)	63.7 (15.0) 65.8 (13.8) 62.1 (15.8)	57.4 (15.6) 59.7 (14.8) 55.5 (15.9)	70.8 (11.9) 71.0 (11.4) 70.7 (12.2)	62.7 (16.7) 63.2 (15.6) 62.3 (17.8)
Age-standardised rate x 10^5^ PY	5.0	42.1	31.7	10.3	21.4	6.0	3.6
Age-standardised rate x 10^5^ PY from 1989 to 2023	3.4 to 4.7	19.5 to 73.1	16.6 to 50.4	2.9 to 22.7	6.5 to 38.1	1.5 to 10.0	2.4 to 4.4
Estimated annual percent change, EAPC	1.0%	4.5%	3.9%	6.6%	4.9%	4.7%	1.5%
Year with 1 JP	2011	2016	2013	2003	2011	1994	2010
EAPC before and after the JP	+2.6% (2.3; 2.9) –2.0% (-2.7; -1.5)	4.9% (4.6; 5.6) 2.8% (-0.8; 4.1)	4.6% (4.0; 5.2) 2.1% (1.8; 2.6)	5.2% (4.9; 5.7) 7.0% (6.7; 7.3)	6.5% (6.1; 7.0) 2.7% (1.9; 3.3)	19.0% (12.5; 31.7) 4.4% (2.3; 7.3)	+3.1% (1.3; 7.5) –1.9% (-8.4; 5.6)

M, mean; SD, standard deviation; PY, person-years; EAPC, estimated annual percent change; JP, joinpoint. The numbers in parentheses are 95% confidence intervals.

**Figure 1.**
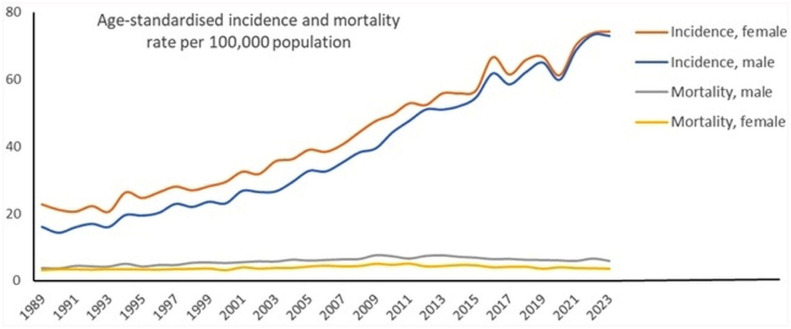
Age-standardised melanoma mortality rates and incidence rates of in situ and invasive lesions combined per 100,000 population in the Netherlands for men and women.

Figure 1 visualises the trends in both mortality and incidence by sex. The melanoma mortality for men and women showed a gradual increase before 2011, and thereafter a slight decrease. Across the same period, much higher melanoma incidence rates were observed, with a sharply rising fourfold increase from nearly 20 to 80 per 100,000 population.

### Trends in rates of specific melanoma types

**Figure 2.**
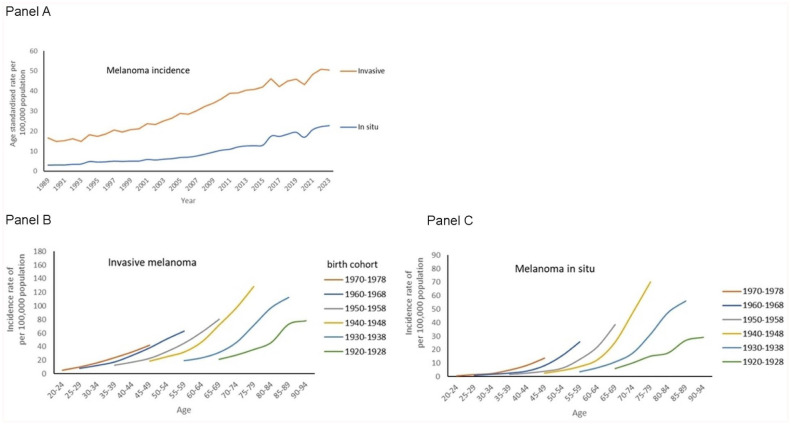
Time trends in age-standardised incidence rate of invasive melanoma and melanoma in situ per 100,000 total population in the Netherlands between 1989 and 2023 (panel A). Details are age-specific incidence rates by birth cohort from 1920–1928 to 1970–1978 for invasive melanoma (panel B) and melanoma in situ (panel C).

For both invasive and in situ melanoma age-standardised rates increased, see Figure 2, panel A. In Table 1, the associated jointpoint calculations for the annual percent change are presented: EAPC = 3.9% vs. EAPC = 6.6%, respectively. After 2013, the EAPC for invasive melanoma was 2.1%, 95% confidence interval (1.8; 2.6). For melanoma in situ we calculated a larger EAPC since 2003, namely 7.0%, 95% confidence interval (6.7; 7.3).

Table 2 presents the age and period-specific incidence rates of in situ and invasive melanoma combined per 100,000 population. In men and women, the rates increased across age categories and calendar periods. In the age-group 20-49 year, rates plateaued from 2009 onwards. In Figure 2, the age-standardised rates of invasive and in situ melanoma are displayed, showing increased curves. The epidemiological curves of the birth cohorts successively born in 1920–1928 to the most recent cohort of 1970–1978 are visualised in panels B and C. Sharply increasing age-specific incidence rates for invasive and in situ melanoma were observed.

**Table 2.** Age and period-specific incidence rates of cutaneous melanoma per 100,000 person-years in the Netherlands between 1989 and 2023 for males (part A) and females (part B) in the Netherlands.

Period / Age	20-49	50-74	75-84	85+
Part A – MALES: Incidence rate of melanoma (in situ and invasive combined) per 100,000 person-years				
1989–1993	9.1	21.8	31.1	31.6
1994–1998	11.0	29.6	46.9	47.0
1999–2003	12.3	37.4	56.2	60.6
2004–2008	15.8	50.9	79.8	80.6
2009–2013	19.5	72.5	120.0	134.1
2014–2018	20.2	99.0	193.5	201.6
2019–2023	18.9	120.3	321.8	307.7
Part B – FEMALES: Incidence rate of melanoma (in situ and invasive combined) per 100,000 person-years				
1989–1993	16.0	27.0	29.0	31.0
1994–1998	19.1	33.9	42.7	37.1
1999–2003	23.2	40.8	48.6	41.7
2004–2008	28.4	52.5	63.2	56.7
2009–2013	34.4	72.2	86.5	80.4
2014–2018	36.8	95.4	119.9	95.2
2019–2023	33.3	118.2	188.7	138.4

**Figure 3.**
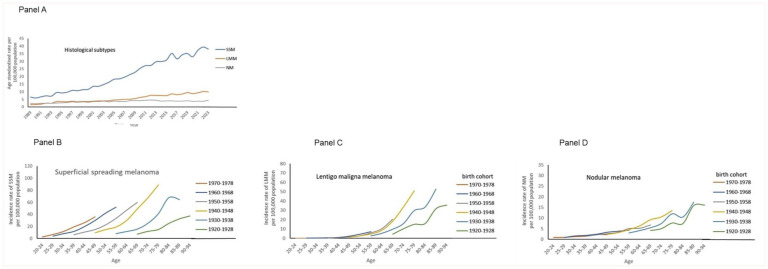
Time trends in age-standardised incidence rate of superficial spreading melanoma, lentigo maligna melanoma and nodular melanoma per 100,000 total population in the Netherlands between 1989 and 2023 (panel A). Details are age-specific incidence rates by birth cohort for the three histological subtypes (panel B to D).

In Figure 3 on histological subtypes, panel A showed elevated levels of the epidemiological curves over time. In panels B to D, the curves were arranged to birth cohort. For SSM, strong elevations were noted towards the younger cohorts. For LMM also a clear birth cohort effect was observed, but hardly for NM with overlapping curves.

### Future trajectory

**Figure 4.**
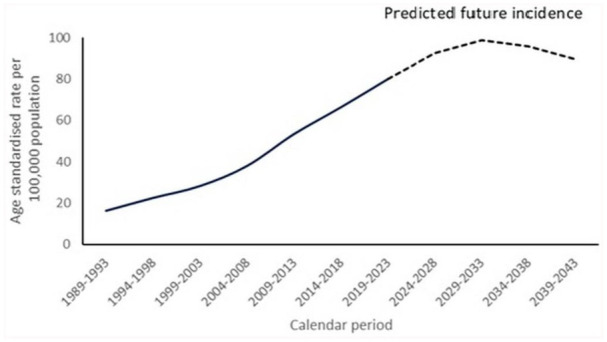
Calculated (1989-2023) and predicted (2024–2043) age-standardised incidence rates per 100,000 total population for all types of melanomas in the Netherlands.

The results on future rates of all types melanoma are given in Figure 4. Following the period and birth cohort patterns of SSM and LMM, after years of growth a peak was predicted around 2030 and a decline towards 2040 (results not presented). The less frequent NM subtype showed steady levels from 2010 onwards at approximately four per 100,000 person-years.

## Discussion

The use of data on histological subtypes of cutaneous melanoma provided new insights into the trajectories of the rising epidemiological curves. In general, excess incidence rates brought about by diagnostic scrutiny and overdiagnosis would be expected in all types, whereas larger numbers of patients caused by increased exposure to ultraviolet radiation are likely to occur in clearly sun-related subtypes.

In this study on 182,987 primary diagnosed patients aged 20 years and over, the age-standardised incidence of melanoma in situ and invasive melanoma combined has been steadily increasing from 19.5 per 100,000 population in 1989 to 73.1 per 100,000 in 2023. For invasive melanoma separately, the estimated annual percent change was 3.9%, and for melanoma in situ 6.6%. For the three major subtypes of SSM, LMM and NM, the annual increase in incidence was 4.9%, 4.7%, and 1.5%, respectively. Furthermore, a birth cohort effect was shown of younger generations being more frequently diagnosed with SSM and LMM than older generations. Such a pattern was almost absent for NM.

The melanoma mortality showed peak years around 2010 and gradually decreasing rates thereafter, which was a confirmation of a previous study.^20^ This is contrasted to the increasing incidence of invasive melanoma. The upward trend for melanoma in situ analysed here was more pronounced, especially in the elderly, yielding additional evidence that a birth cohort effect was present.^15,21^ This means that exposure to ultraviolet radiation and its associated risk of melanoma differs across successive generations. Increased incidence reflecting overdiagnosis from increased skin screening and skin biopsies assumes a period effect from the time early detection is put into clinical practice.

Our findings are in line with international data from the United States and Europe.^22-25^ SEER data from the US showed increasing trends for invasive (EAPC = 3.6%) and in situ lesions (EAPC = 9.5%).^22^ In a European study based on 18 cancer registries, the incidence increased similarly over the 1995–2012 period.^25^ The authors calculated comparable changes averaged over the registries for invasive melanoma (EAPC = 4.0% in men, and 3.0% in women), and melanoma in situ (EAPC = 7.7% in men; 6.2% in women).

For the European countries it was stated that the increases could reflect proportions of the populations received early ultraviolet exposure at a time when no primary interventions, such as public UV-protection campaigns at early age, were available.^16,24,25^ Incidence rates were rising, particularly in White populations, related to increased exposure to ultraviolet radiation from natural sunlight and indoor tanning. A large part of the increase in incidence was for lesions on the limbs and trunk.^25^ Furthermore, the importance of health disparities was addressed, which are widespread in the United States. Numerous studies have documented disparities in melanoma thickness, stage, and site in regard to race and ethnicity, sex and occupation.^22,23^

Most literature addressing histological subtypes are on histopathological features of melanoma, which is crucial to comprehend biology, clinical behaviours and therapeutic responses. Etiological research, however, is being published less frequently. Only recently, Elder and colleagues were able to divide melanomas on the sun-exposed skin by the histopathologic degree of cumulative solar damage of the surrounding skin and the presence of solar elastosis.^26^ But the causal link between solar damage and the SSM and LMM subtypes still deserves more research to truly solidify it. Nevertheless, the presence of solar elastosis found in these subtypes is a good indication that the link is acceptable. Our study showed birth cohort effects for these two subtypes adding further evidence to support the sun damage etiology and explaining the increased incidence, which was probably not the case for NM.^27-29^ As stated above, more articles have been published on the issue of overdiagnosis, but unfortunately without specifications to histology. If overdiagnosis due to the detection of indolent pathological lesions caused by diagnostic scrutiny is a major culprit of increased incidence, then one would expect rising figures for all histological subtypes. We observed distinct patterns for SSM and LMM, but not for NM.

The main limitation of the present study is that we had no access to registered data on biopsy taking and the related information on the extent and assessment of the biopsy specimens. This makes it difficult to gain a complete understanding of increased diagnostic scrutiny across the years and the consequences for under- and overdiagnosis, especially for the different histological types. A second major issue of our study is the descriptive epidemiological nature of the age-period and age-birth cohort analysis. Full age-period-cohort statistical modelling is needed that allows trends to be uniquely separated into the influences of age, period, and birth cohort. Additionally, the incidence rates for the most recent birth cohorts were low and should thus be interpreted with caution.

Numerous association and geographic-temporal correlation studies have been conducted on trends in melanoma incidence.^13,14^ In a scoping review of 28 studies, the degree of overdiagnosis was estimated to range from 29% to 60%.^13^ However, not only empirical data but also study designs and analyses differed largely. This is understandable due to difficulties in obtaining unscreened reference populations, lack of knowledge and data on the natural (pre)clinical course, and the amount of time by which date of diagnosis was advanced by early detection.^30^

Recently, Yuan, et al., evaluated the global burden of malignant skin melanoma from 1990 to 2019 and calculated projections until 2034 using data from 204 Global Burden of Disease countries and territories divided into 22 regions.^31^ For data analysis, they applied a comprehensive age-period-birth cohort framework. The age-standardized incidence rate of malignant skin melanoma varied greatly throughout the world, with Australasia having the highest rate of 43.4 (uncertainty interval, 30.2-57), similar to the Netherlands with 42.1 per 100,000 population a year (Table 1). Unfortunately, no specifications to address the international burden of overdiagnosis were presented.

Also recently, attention to upward trends in cutaneous melanoma was given by the EUROCARE-6 Working Group, analysing 108 cancer registries in 29 EU countries.^32^ In line with our results (Table 2) they found rates to increase with age, being higher in females than in males among 15–39 year olds, and vice versa in elderly males. No details were given to risk factors, histological type or possible overdiagnosis.

## Conclusions

By including histological subtypes of melanoma in trend studies on age, period and birth cohort specific incidence rates, new insights are given that a burden of overdiagnosis exists, but at a lower level than systematic reviews suggest.

In designing future studies to explain trends in melanoma incidence and mortality it is important to define determinants and outcomes and the time relation between these. The relationship should also comprise histological subtypes combined with the standard pathology data on tumour spread, and with Breslow values. The parameters need time-specific information on age, calendar year, and year of birth. Biopsy data are necessary to work out the topic of diagnostic scrutiny. In this way, joinpoint analysis and age-period-cohort modelling will yield relevant details on benefits and harms, and deliver useful instruments for shared-decision making and clinical follow-up.
